# Alterations in expressed prostate secretion-urine PSA N-glycosylation discriminate prostate cancer from benign prostate hyperplasia

**DOI:** 10.18632/oncotarget.20299

**Published:** 2017-08-16

**Authors:** Gaozhen Jia, Zhenyang Dong, Chenxia Sun, Fuping Wen, Haifeng Wang, Huaizu Guo, Xu Gao, Chuanliang Xu, Chuanliang Xu, Chenghua Yang, Yinghao Sun

**Affiliations:** ^1^ Department of Urology, Changhai Hospital, Second Military Medical University, Shanghai 2000433, China; ^2^ Joint Center for Translational Research of Chronic Diseases, Changhai Hospital, Second Military Medical University, Shanghai 2000433, China; ^3^ Institute for Nutritional Sciences, Shanghai Institutes for Biological Sciences, Chinese Academy of Sciences, Shanghai 200031, China; ^4^ State Key Laboratory of Antibody Medicine and Targeted Therapy, Shanghai 201203, China

**Keywords:** prostate cancer, PSA, glycosylation, benign prostate hyperplasia, EPS-urine

## Abstract

The prostate specific antigen (PSA) test is widely used for early diagnosis of prostate cancer (PCa). However, its limited sensitivity has led to over-diagnosis and over-treatment of PCa. Glycosylation alteration is a common phenomenon in cancer development. Different PSA glycan subforms have been proposed as diagnostic markers to better differentiate PCa from benign prostate hyperplasia (BPH). In this study, we purified PSA from expressed prostate secretions (EPS)-urine samples from 32 BPH and 30 PCa patients and provided detailed PSA glycan profiles in Chinese population. We found that most of the PSA glycans from EPS-urine were complex type biantennary glycans. We observed two major patterns in PSA glycan profiles. Overall there was no distinct separation of PSA glycan profiles between BPH and PCa patients. However, we detected a significant increase of glycan FA2 and FM5A2G2S1 in PCa when compared with BPH patients. Furthermore, we observed that the composition of FA2 glycan increased significantly in advanced PCa with Gleason score ≥8, which potentially could be translated to clinic as a marker for aggressive PCa.

## INTRODUCTION

Prostate cancer (PCa) is the most commonly diagnosed cancer and second leading cause of cancer death in men [1]. Prostate-specific antigen (PSA) is a protein that is almost exclusively produced in the prostate. Normally, PSA is confined to intact prostate tissue, and serum PSA level is very low. In disease conditions like PCa, the prostate structure is disrupted and PSA leaks into the circulation, resulting in an elevated serum PSA level. Therefore, serum PSA level has been widely used as a biomarker for PCa detection and progression monitoring. However, serum PSA elevation is not cancer specific; PSA level also increases in conditions such as benign prostate hyperplasia (BPH) or inflammation. With a cut-off level at 4 ng/ml, the sensitivity and specificity of the PSA test are 21% and 91%, respectively [2]. Because of the low sensitivity of the PSA test, the wide use of serum PSA test has resulted in overdiagnosis and overtreatment of indolent PCa [3, 4]. Therefore, biomarkers that could effectively differentiate prostate cancer from benign prostate conditions and provide better guidance in clinical practice are needed.

To improve the specificity of the PSA test, the ratio of free PSA to total PSA (fPSA/tPSA) is commonly used in clinic in addition to the PSA test. The fPSA/tPSA is usually lower in PCa than in BPH. However, no clear cut off value effectively discriminates PCa from BPH. PSA velocity and PSA density were also used as additions to improve the specificity of the PSA test. However, no significant improvements were observed for these methods. [-2] proPSA (also known as P2PSA), a different form of PSA, was found to be specific to PCa. Prostate Health Index (PHI), which combines total PSA, fPSA, and [-2]proPSA, improves the specificity and was recently approved in the U.S., Europe and Australia for PCa diagnosis [5, 6]. Although the PHI test has a relative high sensitivity (85%), the specificity in PCa detection is still low (approximately 45%), its ability to discriminate high-grade PCa from low-grade is even lower (~17%), limiting its broader clinical application [7].

Novel biomarkers have been developed recently, which include prostate-specific membrane antigen (PMSA), α-methylacyl-CoAracemase (AMACR), PCA3, and the TMPRSS2-ERG fusion gene [8, 9]. Among these biomarkers, PCA3 and the TMPRSS2-ERG fusion gene are the most promising in predicting prostate cancer. PCA3 is a prostate-specific gene and it is overexpressed in PCa [10]. A urinary PCA3 test was approved by the U.S. Food and Drug Administration (FDA) for the prediction of PCa after an initial negative biopsy [11]. The TMPRSS2-ETS fusion gene was present in approximately 50% of PCa patients in Western countries [11–13]. Like the PCA3 test, the urinary TMPRSS2-ETS score was also developed to evaluate PCa risk [14]. A combination of urinary PCA3 and TMPRSS2-ERG scores might improve PCa prediction on biopsy [14]. However, PCA3 cannot differentiate high-risk PCa from low-risk and intermediate-risk PCa. Furthermore, the occurrence the TMPRSS2-ERG fusion gene is low in Asian ethnic groups [15]. Its translational benefit for Asian patients is obscure.

Glycosylation alternation is a common feature in cancer development and progress [16, 17]. Serum glycome profile alterations have been demonstrated in breast carcinoma, PCa, lung cancer, and stomach cancer [18–22]. Among all human proteins, approximately 50% are glycosylated [23]. PSA is a glycoprotein with 7% to 8% carbohydrate content [24, 25]. PSA has one N-linked glycosylation site at Asn-69 and one o-linked glycosylation site at Thr133 [26]. The possibility of using different PSA glycan isoforms as biomarkers in differentiating aggressive PCa from BPH has been explored. Fucosylation and sialylation changes in PSA were frequently observed to be associated with PCa development [27–32]. However, these studies were based on cancer cell lines or small patient numbers. The clinical relevance of these studies must be verified using larger patient cohort. Furthermore, the genetic differences among different ethnic groups might also affect the glycosylation profiles in PSA. In this study, we characterized PSA glycosylation profiles for 62 expressed prostate secretion-urine (EPS-urine) samples from Chinese patients, including 32 BPH and 30 PCa patients. We found that most of the PSA glycans from EPS-urine were complex-type biantennary glycans. PSA glycan profiles showed two major patterns. We detected a significant increase of FA2 and FM5A2G2S1 glycans in PCa compared with BPH. Furthermore, we observed that compared with BPH and low-risk/intermediate-risk PCa, the composition of FA2 glycan increased significantly in advanced PCa with Gleason scores ≥8, which could be potentially translated to clinic as a marker for aggressive PCa.

## RESULTS

### Clinical characteristics of the patient population

Micro-grams of PSA are required for purification and characterization of the PSA glycan profile by the ultra performance liquid chromatography-mass spectrometric (UPLC-MS) method, a commonly used method in antibody drug quality control (QC) analysis. However, the concentration of serum PSA in BPH and PCa is usually in the range of 4-100 ng/ml, A sufficient amount of PSA protein from serum for glycan analysis is difficult to obtain. Some studies investigated serum PSA glycome from PCa patients with serum PSA levels greater than 1000 ng/ml [29, 31, 32], which is high-risk PCa and not suitable for early diagnosis purpose. PSA is an abundant protein in prostate fluid and semen plasma. EPS-urine is collected after standard prostate massage (three left-side strokes, three right-side strokes and three middle-sidestrokes) during a digital rectum examination. EPS-urine can be easily collected and is rich in prostate fluid and PSA. The concentration of PSA in EPS-urine could be in the range of 5 to 20 μg/ml (unpublished data), approximately 1000-fold higher than serum PSA [33], making it a good source for PSA purification and glycan profile analysis.

EPS-urine samples were prospectively collected from 62 patients, of which 32 were diagnosed with BPH and 30 were diagnosed with PCa, according to the standard pathological diagnosis procedure. The patients’ ages in BPH group ranged from 49 to 77 years, and in the PCa group ranged from 58 to 75 years. The clinical characteristics of the patients were summarized in Supplementary Table 1. Of the 30 PCa patients, 15 were low-risk and intermediate-risk PCa with a Gleason Score between 6-7, and 15were high-risk PCa with Gleason Score ≥8.

### Purification of PSA from EPS-urine

**Figure 1:**
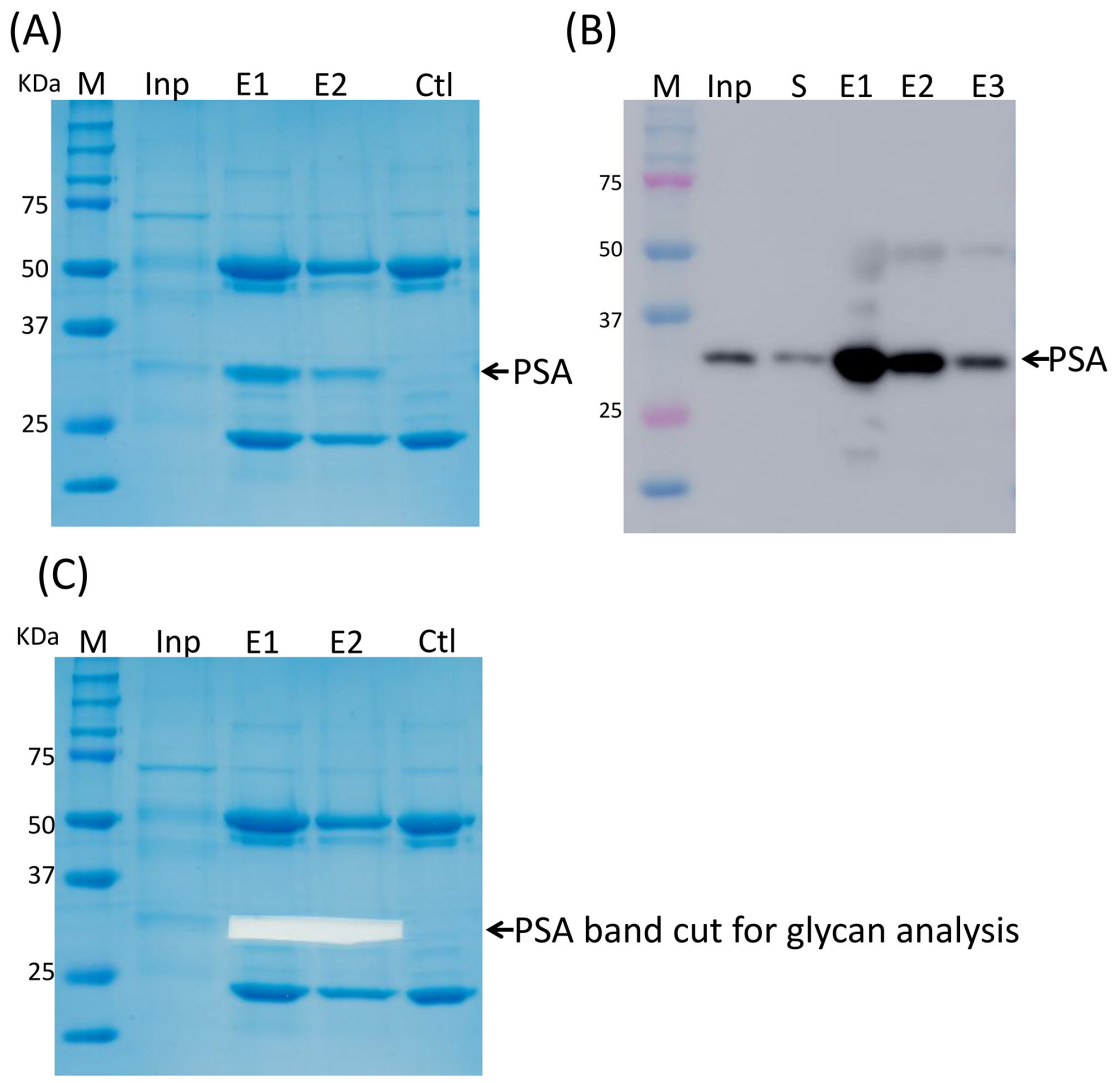
PSA purification by immunoprecipitation **(A)** PSA purified by IP. **(B)** PSA was confirmed by Western blot. **(C)** PSA band was cut off for further glycan profiling analysis.

The PSA content in the samples was evaluated by sodium dodecyl sulfate-polyacrylamide gel electrophoresis (SDS-PAGE) and confirmed by Western blot (data not shown). Anti-PSA immunopurification was used to purify PSA from the EPS-urine samples. Electrophoresis was further applied to separate PSA from other glycoproteins such as antibodies. Figure 1A showed that the purity of the PSA purified by this method was approximately 95%. Purified PSA was further confirmed by Western blot analysis (Figure 1B) and mass spectrum identification (data not shown).

To test whether the immunoprecipitation of PSA was dependent on the PSA glycan, we first removed the PSA glycans utilizing PNGase F before the immunoprecipitation of PSA. PNGase F removed only the N-glycan from PSA under denaturing conditions, but not in non-denaturing conditions (Supplementary Figure 1, Lanes 6 and 9). With the removal of PSA glycans, anti-PSA antibody partially immunoprecipitated PSA under denaturing conditions (Supplementary Figure 1, Lanes 10 and 11), suggesting that the immunoprecipitation could be partially dependent on the PSA glycans.

### UPLC-FLD/QTOF-MS quality control

PSA bands in the SDS-PAGE gel after immuno-purification were excised (as indicated in Figure 1C) and digested with PNGse F in gel to release N-glycans from the PSA. The N-glycans were fluorescently labeled stoichiometrically with RapiFluor, a newly developed fluorescent labeling reagent by Waters Corporation (Milford, MA, USA), which has a higher fluorescence sensitivity and mass spectrometry response. Then fluorescently labeled N-glycans were analyzed by normal phase UPLC and mass spectrometry, which has become the standard method to evaluate the glycan profiles of antibody drugs for quality control.

The QC sample was a mixture of equal volumes of all the 58 samples. **Fluorescence detector (**FLD) and MS data of the QC samples were measured 11 times at the sample running intervals. Glycan profiles of the QC samples were highly reproducible. The maximum deviation of fluorescence retention time was in the range of 0.1 minutes, and the intensity of the FLD peaks were similar and repeatable as they clustered together in the PCA plot (Supplementary Figure 2). In this study, mass spectra data were used in structure assignment and fluorescence data were used to perform quantitative analysis of the glycan peaks.

### Overall N-glycan profiles of PSA

**Figure 2:**
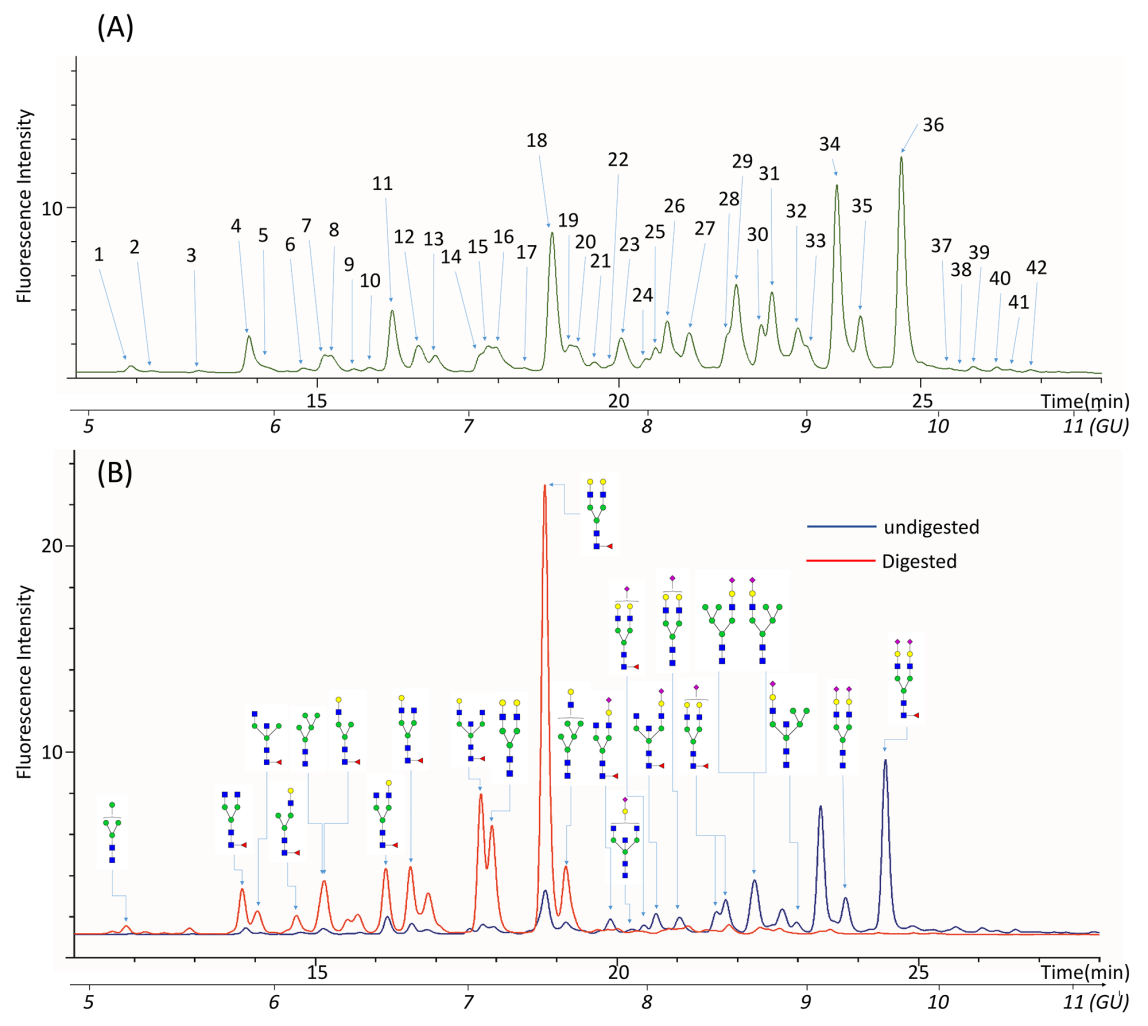
Representative UPLC chromatogram profiles of QC N-glycan samples **(A)** 42 peaks were identified in the glycan profiles of QC samples. **(B)** Glycan profiles before and after digestion with α2-3,6,8,9 Neuraminidase to remove all the sialic acids residues.

The glycan profiles of the QC samples represented the average glycan profiles of all the samples. A typical N-glycan profile of PSA in a QC sample was shown in Figure 2A. The glycan profiles were separated into 42 peaks as indicated (Figure 2A) with glucose unit (GU) values ranging from 5 to11. Mass data for glycans in each peak were collected by electrospray ionization-quadrupole time of flight (ESI-QTOF) mass spectrometry. The major glycan components in each peak were assigned according to their GU values and mass average based on information of known glycans in the RapiFluor-labeled glycans library released in Glycobase 3.2 (https://glycobase.nibrt.ie/glycobase/browse_glycans.action?reportId==36) as well as in the glycans library in GlycoWorkbench [34]. Currently, 177 RapiFluor-labeled glycans with GU values and mass average have been experimentally determined by UPLC-MS and released on the Glycobsase 3.2 website. Based on the GU values and mass average information in the database, the structures of glycans in 28 of 42 PSA glycan peaks can be assigned as shown in Supplementary Table 2. The results showed that most of the PSA N-glycans were complex-type biantennary oligosaccharides, which is consistent with previously reported structures [25, 30, 32, 35]. The assignments of the glycans were further validated by exoglycosidase digestion. According to the assignments, glycans in peaks 20 to 42 were sialylated. When the glycans were digested by α2-3,6,8,9 Neuraminidase A, which removes all the sialic acids at the terminal of glycans, glycan peaks from 20 to 42 disappeared (Figure 2B). This outcome validated that the assignments in peaks 20 to 44 were sialylated glycans. Because some of the peaks in 22 to 42 were not assigned on the basis of the libraries we used, we could not calculate the exact composition of the sialylated glycans that were proportionally converted to their non-sialylated counterpart glycans after Neuraminidase A digestion.

### N-glycan profiles characterization of PSA from PCa and BPH

The FLD and MS information were collected for EPS-urine samples from 32 BPH and 30 PCa patients. The glycan profiles of the 62 samples were aligned with profiles of the QC samples and the peak numbers were assigned as projected in the QC glycan profiles. Because the absolute FLD intensities of glycan peaks were different between samples, the relative percentages of peak areas were calculated, which represented the composition of glycans within each peak in a sample. Therefore, the compositional data rather than absolute quantity data were used for further analysis. The results were summarized in Supplementary Table 3.

**Figure 3:**
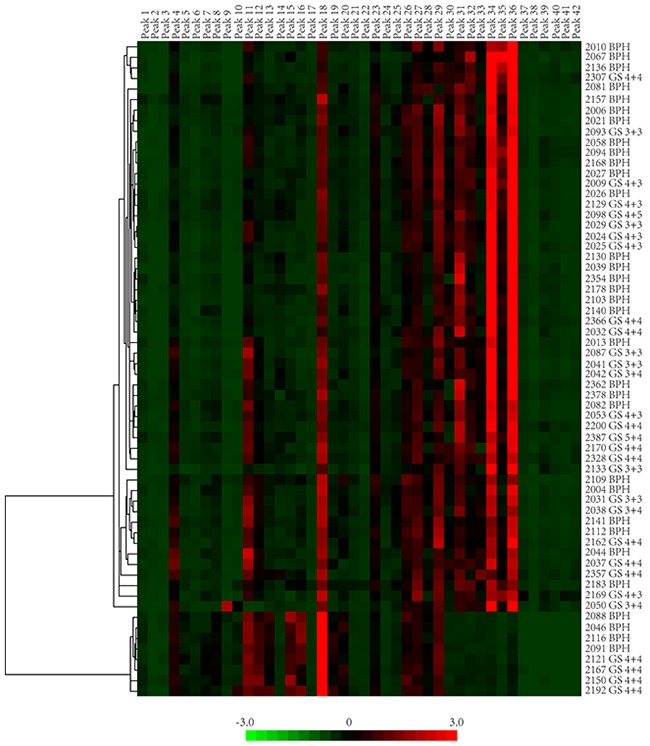
The heat map presentation of glycan profiles of 62 samples PSA glycan peak composition of BPH and PCa were hierarchically clustered by Cluster 3.0 and visualized in the in heat map by JavaTreeview. Two major patterns could be identified in these 62 glycan profiles.

To directly visualize and compare the glycan profiles of all 62 samples, the compositional data of all the glycan peaks from BPH and PCa groups were clustered and visualized in a heat map (as shown in Figure 3). From the heat map clustering, we observed two major patterns in the PSA glycan profiles. Peaks 12-16 and 30-36 were the major groups of peaks that could differentiate all the glycan profiles into two patterns: pattern 1 represented N-glycan profiles that consisted of a low composition of peak 12-16 but a high composition of peaks 30 to36; pattern 2 had a high composition of peaks 12 to16 but a low composition of peaks 30 to 36. According to the assignment, peaks 12 to15 mostly contained core-fucosylated biantennary glycans, and peaks 30 to36 mostly contained di-sialylated biantennary glycans. The glycan profiles in the BPH group were very diverse and contained both patterns (pattern 1 and 2). Pattern 2 presented in high-risk PCa with GS≥8, but not in low-risk/intermediate-risk PCa. Furthermore, pattern 2 represented a group of glycan profiles that contained low levels of di-sialylated glycans: 4 in 15 (26.67%) of the high-risk PCa with GS≥8, and 4 in 32 of the BPH group (12.5%) had this type of glycan profile, suggesting that low levels of di-sialylated glycans can be a common feature in high-risk PCa. However, this possibility must be validated in a larger sample cohort.

**Figure 4:**
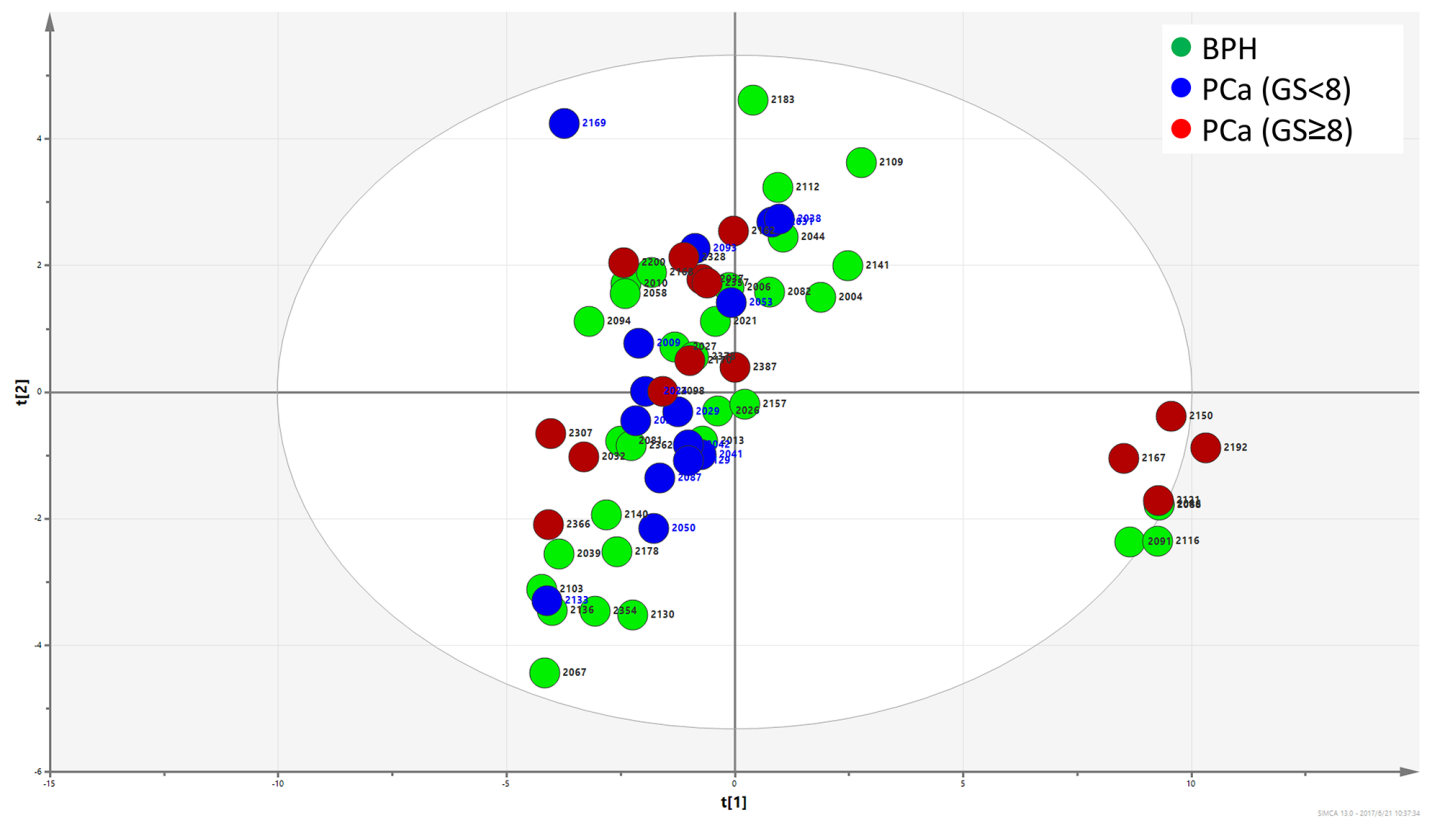
Principle component analysis of FLD data The distribution of glycan profiles from BPH and PCa were scattered, and no obvious separation of BPH and PCa was observed.

Principle component analysis (PCA) was utilized for an informative statistical analysis of the glycan profiles. A plot of the scores of PCA1 and PCA2 were illustrated in Figure 4. Like the two patterns identified in heat map clustering, PCA analysis also grouped all the glycan profiles into two distinguishable sets. The right-side set consisted of 4 BPH samples and 4 high-risk PCa samples with GS≥8, which was represented in pattern 2. The left-side set consisted of all three groups of samples including BPH, PCa GS<8 and PCa GS≥8. However, there is no distinct separation between BPH and PCa groups in the PCA plot.

### Glycans significantly altered in PCa

**Figure 5:**
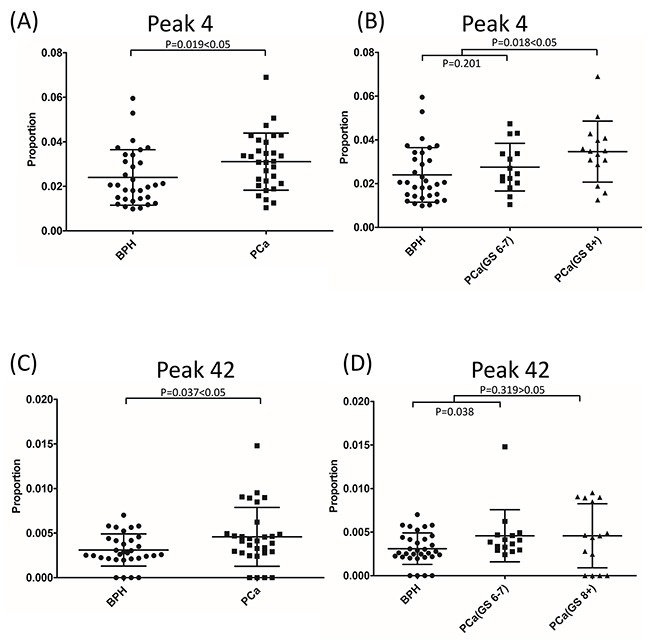
Glycan peak 4 **(A)** and peak 42 **(C)** showed significant difference between BPH and PCa. The composition of peak 4 **(B)**, but not peak 42 **(D)**, increased with the progression of PCa.

**Figure 6:**
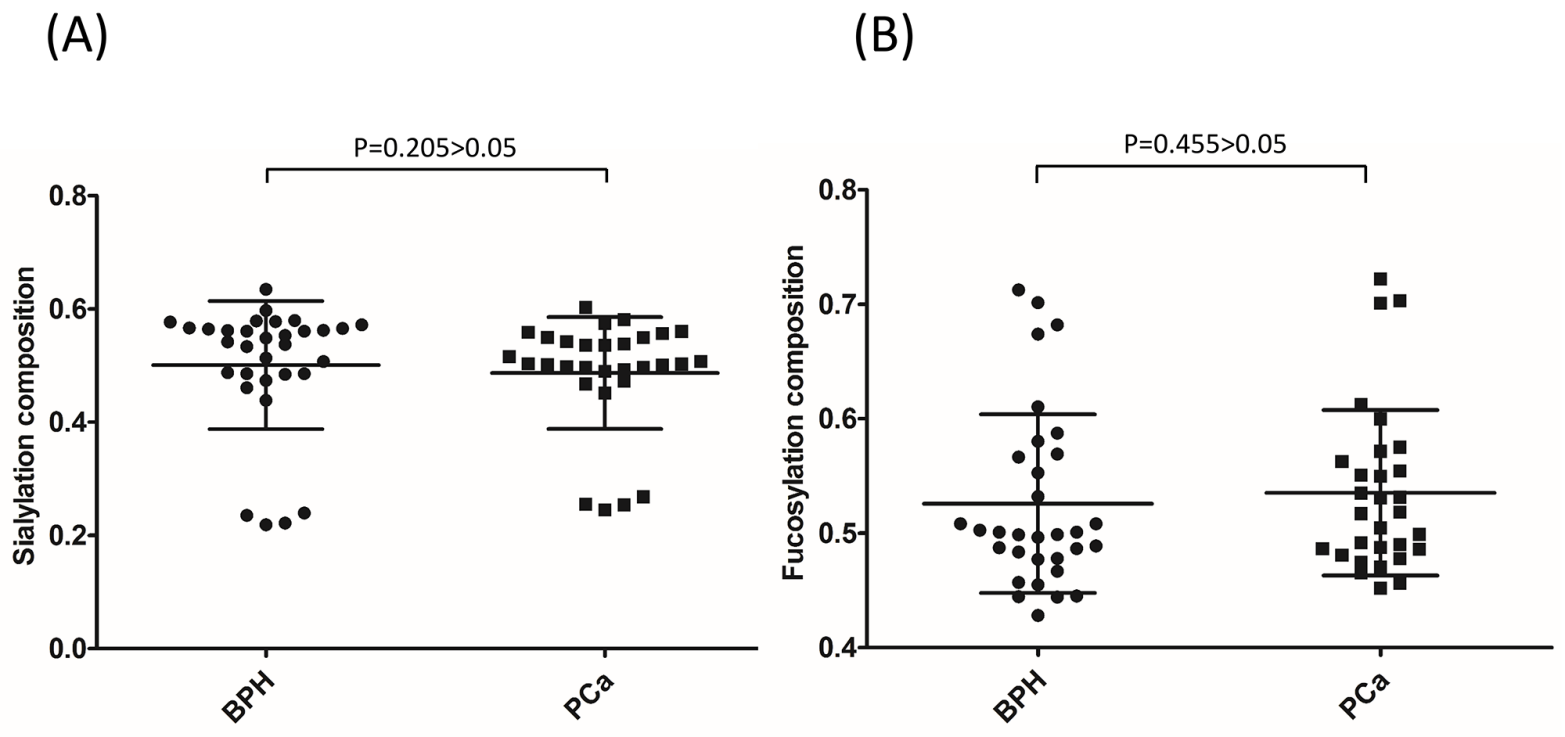
PSA sialylation **(A)** and fucosylation **(B)** content were not significantly different between BPH and PCa.

We analyzed if single peaks were significantly altered in PCa groups when compared with BPH groups. Generally, no peaks in particular belonged to BPH or PCa patients. However, the composition of several peaks was significantly different between BPH and PCa. Statistically, the composition of peaks 4 and 42 increased significantly in PCa when compared with BPH (*P*=0.019 and *P*=0.037, respectively) (Figure 5A and 5C). According to the assignment, the major component in peak 4 was FA2 (also named as G0F), the core-fucosylated biantennary glycan. The major glycan component in peak 42 was FM5A2G2S1. Furthermore, the composition of peak 4 increased significantly with the progression of PCa (*P*=0.018). However, this increase was not observed in peak 42 (*P*=0.319) (Figure 5B and 5D). Sialylation and fucosylation content were calculated in BPH and PCa. No significant difference was observed between these two groups (Figure 6A and 6B).

## DISCUSSION

PSA is a prostate-specific glycoprotein. PSA screening for PCa has been widely applied in clinic. However, the lack of specificity and sensitivity of the PSA test has led to over-diagnosis and over-treatment of PCa. Alteration in glycosylation process is a common feature in cancer development [16, 17]. Evaluating the glycan profile changes of PSA might improve the specificity and sensitivity of PSA screening for PCa. Saldova and colleagues found that in serum N-glycome, the core-fucosylation and alpha2-3 sialylation were significantly increased in PCa patients when compared with BPH patients [20]. However, whole serum proteins contained glycoprotein information from all the organs and tissues. The glycosylation profile alteration of all the serum proteins might indicate the presence of cancer, but cannot give information about the location of cancer. Investigation of the glycan profile evolution of organ-specific glycoproteins might solve this problem.

Previous studies explored the PSA glycan profiles in PCa patients, BPH patients and healthy control subjects [36]. However, the genetic background among ethnic groups is significantly different, the glycosylation pattern could also be different. Currently, only one Chinese group evaluated the urinary PSA glycan profiles in a Chinese population by the use of liquid chromatography-tandem mass spectrometric (LC-MS/MS) method [37]. As suggested in this study by Hsiao et al., the urinary PSA levels can be very low, depending on the collection time. Therefore, a high-sensitivity glycan detection method is required. The study analyzed the glycopeptides of PSA and identified eight different glycans in a pooled sample and selected six of them for further analysis in individual samples. We utilized the EPS-urine samples, an easily collectable resource rich in prostate fluid and PSA content, and extensively characterized PNGase F-released glycan profiles of PSA for 32 BPH and 30 PCa patients and identified 28 PSA glycans. According to the Association of Biomolecular Resource Facilities (ABRF) glycoprotein research in a multi-institutional study [38], glycopeptides and PNGase F methods usually give comparable results. Both studies showed that most of the PSA glycans were biantennary glycans, which is in agreement with previously reports [25, 30, 31, 35]. The eight glycans identified in Hsiao's study [37] were also identified in our study. Furthermore, we identified more glycans in different configurations and provided extensive glycan profiles of PSA in a Chinese population. This advance in PSA glycan profiling is likely the result of the higher PSA content in the EPS-urine samples and higher sensitivity of the RapiFluor-labeled UPLC-MS method. We also compared glycans identified in our study with the ones in the ABRF study, there were 14 glycans identified in both studies [38]. Although the antibody we used to immunoprecipitate PSA was partially dependent on PSA glycans, as shown in Supplementary Figure 1, it did not introduce significant biases in the glycans it selected for the analysis, considering the similar glycan profiles we characterized as in other studies.

PSA fucose and sialylation changes and different types of sialylation linkage were associated with PCa in previous researches [27–31, 39, 40]. Peracaula et al. showed that in comparison with seminal plasma PSA, PSA in LNCaP cells has a higher fucose content and a lower degree of sialylation [28], whereas Tabares et al. showed that PSA glycosylation profiles in serum was significantly different from the profiles in cell lines [32]. From seven PCa patients and one BPH patient, Huber et al. observed that the total sialylation levels of serum PSA were lower in PCa patients than in BPH patients [27]. Sarrats et al. observed the same phenomena in two PCa patients [29]. However, Li et al. reported an elevation of sialylated glycans in cancer tissues when compared with non-cancerous tissues [39]. Because PSA glycosylation was not altered after being released into the serum from prostate tissue [27, 31], the controversy of these studies could likely be to the result of sampling bias from limited sample numbers. In our study with 32 BPH and 30 PCa patients, we observed that the distribution of PSA glycan profiles in BPH and PCa were scattered (Figure 4). The BPH and PCa patients had both patterns of PSA glycan profiles (pattern 1 and 2); whereas high-risk PCa patients, but not low-risk/intermediate-risk PCa patients, had pattern 2 glycan profiles (Figure 3). Pattern 2 represented a type of PSA glycan profile that had low sialylation content (Figure 3). We observed that 26.67% of high-risk PCa with GS≥8, but only 12.5% of BPH patients, had this type of low sialylation glycan profiles. This finding suggested a difference in sialylation among BPH, low-risk/intermediate-risk PCa and high-risk PCa. This difference could indicate that the sialylation/desialylation balance is changed in high-risk PCa. However, this possibility must be validated in a larger patient cohort. If true, the function of sialylation in cancer development and progress is worth investigating. We did not observe a significant difference in total sialylation or fucosylation content in PSA between BPH and PCa (Figure 6)

.

When individual peak difference between BPH and PCa patients was analyzed, no peak in particular belonged to BPH or PCa, (Figure 3). However, we determined that FA2 and FM5A2G2S1, the major glycan components in peak 4 and peak 42, increased significantly in PCa when compared with BPH. Moreover, we observed that the percentage of FA2 increased with the progression of PCa. FA2 composition was not significantly different between BPH and low-risk PCa (GS<8) (*P*=0.201), but it was significantly higher in high-risk PCa (GS≥8) when compared with BPH and low-risk PCa (GS<8) (*P*=0.018) (Figure 5B). A similar phenomenon was observed in a study by Llop et al. [41]. When they analyzed PSA core-fucosylation and sialylation content from 29 BPH patients and 44 PCa patients, they found that α2,3-linked sialic acid content was similar in BPH and low-risk/intermedia-risk PCa, but significantly higher in high-risk PCa. Therefore, the content of FA2 and α2,3-linked sialic acid can potentially be used to effectively distinguish high-risk PCa from BPH and low-risk/intermedia-risk PCa. FA2 is a common core of glycans during glycan synthesis. The FA2 levels also increased in ovarian cancer patients when compared with healthy controls [42]. FA2 in combination of other glycans was significantly increased in lymph node-positive breast cancer patients [43]. An aberrant glycosylation process is common in cancer development. However, the underlying mechanism remains to be elucidated. Recently, Wang et al. reported that under glucose deficiency condition, the O-GlcNAcylated fumarase promoted tumor growth by interrupting its interaction with a transcription factor ATF2 [44]. Alterations in gene expression or post-translational modification of enzymes leading to different enzymatic activities of glycotransferases have been shown to be responsible for the different glycan profiles in cancer tissues [45–47]. The observation that FA2 levels increased in PSA glycosylation profiles in high-risk PCa suggested that the levels or activities of fucosyltransferases might have been changed during the PCa progression. Therefore, FA2 content accumulated during this process, leading to increased FA2 levels. However, the detailed molecular mechanism underlying this process must be investigated in future studies.

In clinic, unlike high-risk/aggressive PCa, low-risk/indolent PCa usually grows slowly and requires no treatment. However, in practice, it is a challenge to distinguish indolent PCa from aggressive PCa, leading to overtreatment of indolent PCa. Biomarkers that can effectively distinguish aggressive PCa from indolent PCa would be highly valuable. To address this need, Irshad et al. found the expression of three genes (FGFR1, PMP22, and CDKN1A) were upregulated in indolent PCa tissues but downregulated in aggressive PCa tissues. Therefore, they proposed that this three-gene panel could serve as a biomarker for indolent PCa [48]. In our study, the FA2 level increased significantly in high-risk PCa, but not in low-risk PCa. Therefore, the composition of FA2 could potentially be a good marker for high-risk PCa. However, this observation and the mechanisms underlying it need to be validated in a larger patient cohort in future studies.

## MATERIALS AND METHODS

### Expressed prostate secretions (EPS)-urine samples collection

EPS-urine samples were prospectively collected from 62 patients at Changhai Hospital, Shanghai, China, following the standard operating procedures of the hospital Ethics Committee. Of these 62 patients, 32 were diagnosed with BPH and 30 with PCa. The patient information were summarized in Supplementary Table 1. Patient identification information was removed and unavailable to investigators to protect the patients’ privacy. All EPS-urine samples were collected after a standard massage of the prostate gland (three strokes each to the left side, right side and middle side) during the digital rectum examination before prostate biopsy. The EPS were then collected in 7 to10 ml of voided urine. The EPS-urine samples were centrifuged at 1400x rpm to remove cell pellets and at 14,000x rpm to remove sediments. Supernatant of the EPS-urine samples was then aliquoted and stored at -80°C until analysis.

According to the Gleason Scores (GS), which is a predictive value of the prognosis of PCa, PCa patients were further divided into two groups: GS<8 and GS≥8. PCa with GS≥8 is usually more aggressive and has a worse prognosis.

### Purification of PSA by immunoprecipitation

The presence of PSA in EPS-urine samples was first examined in SDS-PAGE. PSA was confirmed by Western blot utilizing anti-PSA antibody (Santa Cruz Biotechnology, sc-7638) and mass spectrum identification. EPS-urine samples with clear PSA bands were used for PSA purification and subsequent glycan profile analysis. In this study, PSA was purified as follows: first, PSA was captured by the immunoprecipitation method using anti-PSA antibody. The captured PSA were further separated from impurities in SDS-page. The 1ml EPS-urine samples were first precleared with 100 μl of protein A/G beads (Santa Cruz Biotechnology, sc-2003) at 4 °C for 2 hours to remove non-specific protein A/G binding proteins. Next, 40 μg of monoclonal anti-PSA antibody (Hytest Ltd, catalog no. 4P33-8A6) and 100 μl of protein A/G (Santa Cruz Biotechnology, sc-2003) beads were added to the sample and incubated at 4 °C overnight to allow the capture of PSA. The beads were collected and washed three times with 1 ml 1x PBS buffer. The immunoadsorbed PSA were eluted three times with SDS-PAGE loading buffer, and then, the eluted PSA was loaded onto SDS-PAGE to further separate PSA from impurities by electrophoresis.

### N-glycans analysis

### N-glycans release from SDS-PAGE band

The analysis of PSA N-glycan profiles was performed according to the previously described procedures [49]. Briefly, a gel band containing PSA was excised from SDS-PAGE gel, washed with ddH_2_O, and frozen at -20°C for approximately 30 minutes. The band was cut into small pieces and treated with PNGase F (New England Biolabs Inc., catalogue no. P0704S) overnight to release the N-glycans from PSA bands. N-glycans were further extracted three times with 100% acetonitrile (ACN), washed with ddH_2_O, and then lyophilized and stored at -80°C until fluorescence labeling.

### RapiFluor labeling of glycans

Extracted N-glycans were fluorescently labeled with RapiFluor by use of a GlycoWorks RapiFluor-MS N-Glycan Kit from Waters Corporation (catalogue no. 176003606) according to the product instructions. Excess RapiFluor was removed by a hydrophilic interaction chromatography (HILIC) Phytip column (PhyNexus, catalogue no. PTR 91-10-09). RapiFluor-labeled N-glycan solution was freeze-dried, and then dissolved in 22.5% ddH_2_O/25% DMF/52.5% ACN solution for UPLC-MS analysis.

### Exoglycosidase digestion of glycans

Aliquots of Rapifluor-labeled N-glycans were digested with α2-3,6,8,9 Neuraminidase A (New England Biolabs Inc., catalogue no. P0722L) to remove all the sialic acids. Exoglycosidase digested glycans were cleaned by Phytip column (PhyNexus, catalogue no. PTR 91-10-09), freeze-dried and dissolved in 22.5% ddH_2_O/25% DMF/52.5% ACN solution before UPLC-MS analysis.

### Ultra-performance liquid chromatography and mass spectrometry analysis

RapiFluor-labeled N-glycans were separated by ultra-performance liquid chromatography (UPLC) coupled with a fluorescent detector (FLD) on an Agilent 1290 Infinity UPLC instrument (Agilent, Santa Clara, CA). The separation was based on hydrophilic interaction chromatography (HILIC) using a bridged ethane-silicon hybrid (BEH) Glycan column (2.1×150 mm, 1.7 μm particles) (Waters Corporation, catalogue no. 186004742). Mobile phase A was 100% acetonitrile, mobile phase B was 50mM ammonium formate, pH4.4. Column temperature was set to 60 °C. An injection of 20 μl of RapiFluor-labeled N-glycan sample prepared in 22.5% ddH_2_O/25% DMF/52.5% ACN was used throughout. A 35-minute method was used with a linear gradient of acetonitrile from 75% to 54% at 0.4 ml/min. Fluorescence was measured at a wavelength of 445 nm with an excitation wavelength at 265 nm. The system was calibrated with an external standard of RapiFluor-labeled detran calibration ladder (Waters Corporation, catalogue no. 186006841) as well as an internal standard composed of an equal-volume mixture of all the samples.

An ESI Q-TOF-MS(Agilent, catalogue no. G6540A,) was interfaced with the UPLC after the fluorescent detector. The QTOF-MS system was equipped with Dual Agilent Jet Stream ESI. The MS system was set in positive ion mode (ESI+). Nitrogen was used as desolvation gas. The operating conditions for the mass spectrometer were: source temperature 325°C, desolvation gas flow 10 L/min, sheath gas temperature 400°C, and sheath gas flow rate 11 L/min. The scan source parameters of mass spectrometry were: capillary voltage 3500 V, nozzle voltage 500V, and mass range m/z 100 to 3200. The FLD-MS data were acquired automatically by MassHunter Acquisition System software (B.06.00, Agilent).

### Glycan qualitative and quantitative analysis

RapiFluor-MS Dextran calibration ladder (Waters Corporation, catalogue no. 186007982) was used to calibrate a glycan BEH amide column between runs. Glucose unit (GU) values and retention time of the dextran ladder peaks were fitted by a fifth-order polynomial curve as previously described (GU=s+aT+bT^2^+cT^3^+dT^4^+eT^5^). The GU values for glycan peaks from the samples were extrapolated from the standard curve.

The fluorescence peaks were named sequentially (peak 1 to peak 42) and the GU values for each peak were calculated. Mass spectra data were extracted for every fluorescence peak. Glycan structures in each peak were assigned on the basis of the GU values and mass according to the experimental glycobase in NIBRT (http://glycobase.nibrt.ie/) and glycan libraries in GlycoWorkbench Version 2.1 [34] (Supplementary Table 2).

The area under the peak represents the relative quantities of each glycan. Considering that the absolute quantities of the injected N-glycan sample were not equal, the relative quantities (also known as composition quantities) of glycans were calculated as the percentage of each peak area within all the peak areas of that sample. The sum of all peak areas was set as 1 or 100% (Supplementary Table 3).

### Data analysis

Principle component analysis (PCA) was performed by Umetrics Simca Version 13.0 software (Umetrics, Ascot, U.K.), allowing the direct visualization and clustering of multivariate information. PSA glycan peak composition of BPH and PCa groups were also hierarchically clustered, and visualized in the heat map by Cluster 3.0 and JavaTreeview. The compositional data of each glycan peak among different groups was statistically analyzed by application of the Wilcoxon-Mann-Whitney U test. The difference between two groups was considered statistically significant when *P*-values were less than 0.05.

## SUPPLEMENTARY MATERIALS FIGURES AND TABLES








