# Supplementary material for: Alterations in expressed prostate secretion-urine PSA N-glycosylation discriminate prostate cancer from benign prostate hyperplasia
Source: Oncotarget. 2017 Aug 16;8(44):76987–99. doi: 10.18632/oncotarget.20299 (PMC5652757; doi:10.18632/oncotarget.20299)
Supplement: Supplementary file 1 [file oncotarget-08-76987-s001.pdf]

Alterations in expressed prostate secretion-urine PSA N-glycosylation discriminate prostate cancer from benign prostate hyperplasia

SUPPLEMENTARY MATERIALS

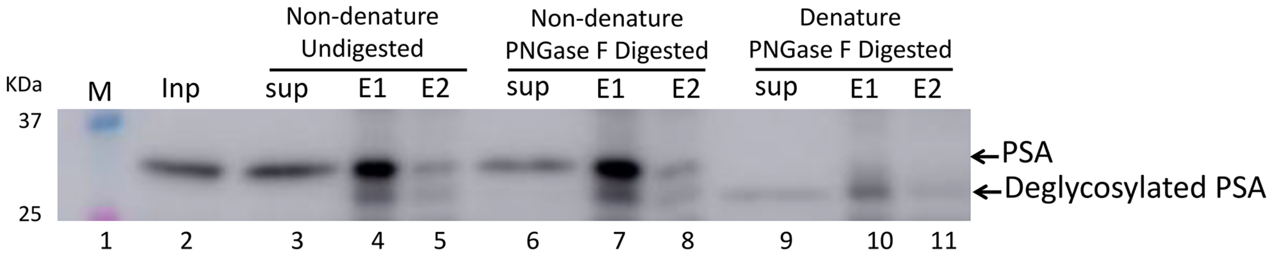

Supplementary Figure 1: Immunoprecipitation of PSA under different conditions. To test whether the immunoprecipitation of PSA is dependent on the glycan of PSA, we first removed the glycans of PSA by use of PNGase F before the immunoprecipitation of PSA. PNGase F only removed the N-glycan on PSA under denaturing condition (Lane 9). Without the PSA glycan, anti-PSA antibody could partially immunoprecipitate PSA under denaturing conditions (Lane 10 and 11).

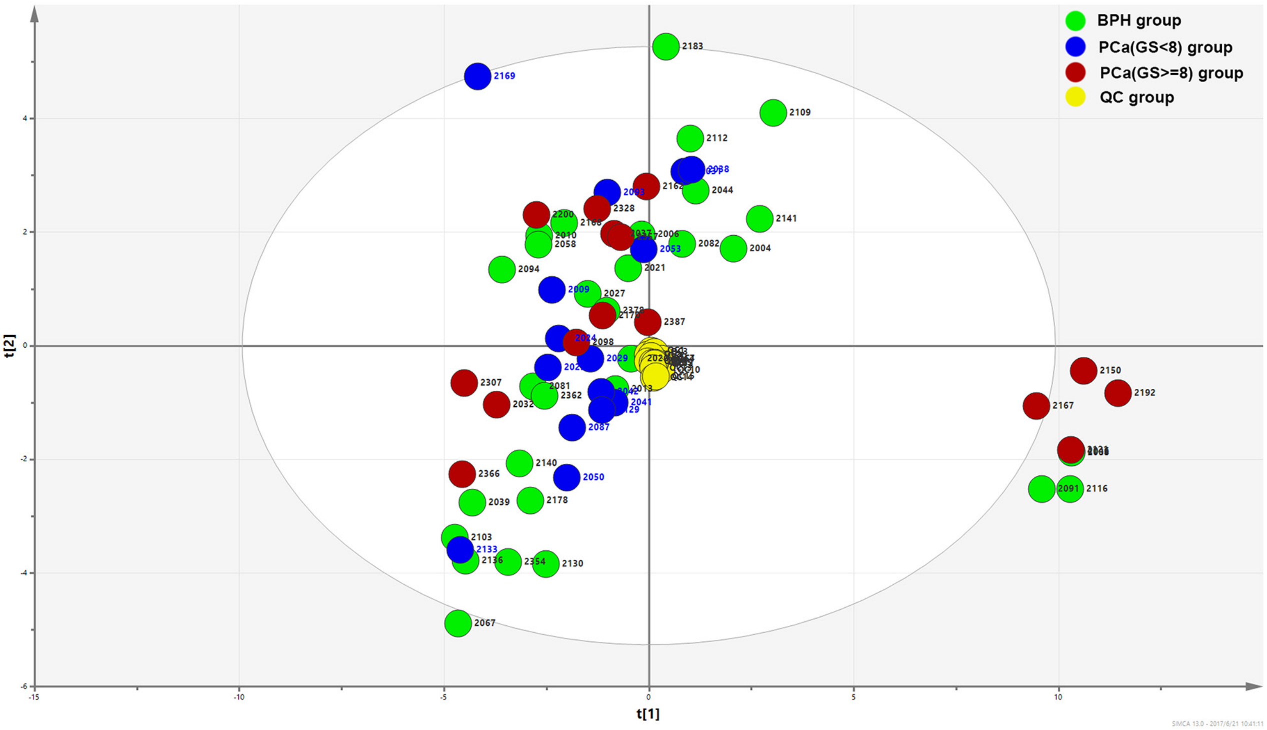

Supplementary Figure 2: Principle component analysis of QC FLD data. The 11 QC data clustered together near the origin of the plot, indicating the good reproducibility of the QC data.

Supplementary Table 1: Patient information.

See Supplementary File 1

Supplementary Table 2: Summary of PSA N-glycan assignment.

See Supplementary File 2

Supplementary Table 3: Summary of PSA N-glycan compositional data.

See Supplementary File 3
